# Radiation therapy students’ perceptions of their learning from participation in communication skills training: An innovative approach

**DOI:** 10.1002/jmrs.200

**Published:** 2016-11-12

**Authors:** Gay M. Dungey, Hazel A. Neser

**Affiliations:** ^1^ Department of Radiation TherapyUniversity of OtagoWellingtonNew Zealand

**Keywords:** Awareness, communication, learning, radiation therapy, simulation training

## Abstract

**Introduction:**

Communication skills training has been progressively integrated into the Bachelor of Radiation Therapy programme in New Zealand throughout the last 3 years. This innovative study aimed to explore students’ perceptions of their learning from participation in communication skills workshops. The purpose was to expose students to a variety of common clinical situations that they could encounter as a student radiation therapist.

**Methods:**

Common scenarios from the radiation therapy setting were developed, using trained actors as a standardised patient, staff member or member of the public. Students were briefed on their scenario and then required to manage their interactions appropriate to its context. A staff member and peers observed each student's interaction via a digital screen and assessed the student's performance in six key skills. Each student was video recorded so that they could review their own interaction. Verbal and written feedback was given to each student. Students evaluated their experience using a 5‐point Likert scale.

**Results:**

Quantitative and qualitative data were collected from 116 of 150 students who consented to participate. Three main themes emerged from the data: the value of learning from peers; preparation for the clinical environment; and the ability to self‐reflect. The quantitative data indicated that students’ perceptions of the tool are positive and an effective learning experience.

**Conclusions:**

Students’ perceptions of participation in the communication skills workshops, with the integration of trained actors, are positive and students perceive the scenarios to be helpful for their learning. Opportunities are indicated to further develop of students’ ability to self‐reflect.

## Introduction

The Bachelor of Radiation Therapy (BRT) is a national 3‐year, full time programme, which is delivered by the University of Otago, Wellington. Radiation therapists in are employed in six regional Departments of Radiation Oncology across New Zealand, and three private hospitals. The programme entails a combination of academic and clinical components across each year. Student cohorts are primarily 18–30‐year‐old females, with approximately 11% being male.

Communication skills workshops, modelled on simulation, have recently been integrated into the curriculum, with a focus on professional interactions with colleagues, patients and general public as per the Medical Radiation Technologists Board's professional Code of Ethics.^1^ This is a novel approach for training of radiation therapists in New Zealand. Simulation is a dynamic process that creates real‐life situations as well as facilitates the active engagement of students in order to reduce the theory‐practice gap through repetition, feedback, evaluation and reflection, without exposing patients to risk.^2^, ^3^ There have been no formal investigations about the value of communication skills workshops/clinical simulations in the radiation therapy arena. However, simulation has been long incorporated into medical, nursing and allied health professional training to improve patient safety, focussing on reproduction of anatomical regions or clinical tasks, along with more complex human interactions, such as communication and teamwork skills, social and emotional competence.^3^, ^4^, ^5^, ^6^, ^7^ Simulation needs to reflect meaningful, authentic situations in which medical services are delivered to enable the suspension of disbelief.^2^, ^8^

*Fidelity* is, therefore, a key aspect of any simulation; this is the degree to which the appearance and behaviour of the simulation are faithful to that of the real situation. Simulation exists on a continuum of low to high fidelity, but should always be authentic. Authenticity allows for more effective student learning through suspension of disbelief.^3^ Using trained actors enhances authenticity because they can portray different levels of emotional distress, along with social contexts, in a realistic and consistent manner.^8^, ^9^, ^10^, ^11^ *Low fidelity* simulation activities provide opportunities for students to discuss what they would do, such as case based stories, or practise discrete tasks. *High fidelity* refers to more complex activities that immerse students in the situation, and so the activity must be believable and true to the task environment. The goal of the training should therefore guide the simulation's fidelity, within authentic contexts.

The benefits of simulation have been widely acknowledged to improve student performance in managing a range of clinical situations in a safe environment.^2^, ^4^, ^12^, ^13^, ^14^ However, LeBlanc^5^ cautions about the hidden danger of simulation: it may increase students’ self‐confidence and perceived abilities without actually developing them because students may cease to practise skills in the belief that they have reached an acceptable level of competency. Evidence is also conflicted about whether there is any transfer of learning from simulated to clinical settings, including the radiation therapy setting.^15^ This aspect of student learning warrants further research.

The value of simulation is that it is experiential learning, espoused by Kolb as the way to transform new experiences via a cycle of ‘do, observe, think, and plan’ to develop knowledge.^16^ Simulation offers hands‐on practice with a real person/situation, engaging active learning through practice and discussion via group debriefing and shared problem‐solving.^17^ This facilitates the development of higher‐order thinking skills like analysis, synthesis and evaluation and enables students to identify their own strengths and weaknesses through self‐review^3^, ^12^ An integral aspect of learning gained from simulation is through self‐reflection.^12^ According to Bandura,^18^ self‐reflection is one of the core components of learning, along with intentionality, forethought, and self‐regulation. Therefore, simulation could be a helpful tool to develop radiation therapy students’ metacognitive skills required for reflection on their performance.

Effective debriefing improves learning, skill development and reflection after the simulation experience.^11^, ^13^, ^17^, ^19^, ^20^ According to Dannefer et al.,^21^ students report that the process of giving feedback to peers is both challenging and stimulating but overall invaluable. Feedback from actors is also helpful because it can provide insight about the impact of the student's actions from the perspective of patient, colleague or member of the public, which is not generally shared with health professionals in clinical settings. Debriefing aligns with Vygotsky's^22^ concept of the ‘zone of proximal development’ (ZDP), in which learning occurs through active problem solving and discussion with peers and teachers in order to make links between new ideas and current understanding. However, what debriefing models, whether peer‐led, teacher‐led, or self‐review, are the most effective for learning to occur are yet to be determined by research.^23^

The aim of this study is to explore radiation therapy students’ perceptions of their learning from participation in communication skills workshops. The purpose of these workshops is to expose students to a variety of common clinical situations that they could encounter. Noting that, the workshops are focused on interpersonal interactions, not specific technical skills of radiation therapy, so there is no single correct way to manage interactions effectively.

## Methodology

All students across each year level of the degree programme were required to participate in the simulated clinical scenarios as part of their formative assessment in the healthcare communication academic papers. Qualitative data were obtained from student written self‐reflections on their perceptions of their learning from participating in these. Quantitative data were collected from a survey of the students’ experience of the learning tool. Analysis was conducted on the data of consenting students.

### Study participants

Following ethical approval from the University of Otago Ethics Committee (reference D13/226) students were asked to consent for their data and self‐reflection forms to be used for the current study. 116/150 undergraduate radiation therapy students agreed to participate (77.3% response rate). Table 1 highlights the demographics of the participants.

**Table 1:** Participant characteristics

	*N*	%
Gender		
Female	104	89.7
Male	12	10.3
Ethnicity		
NZ European/Pakeha	82	70.7
Maori	9	7.8
Pasifika	9	7.8
Asian/Other	16	13.8
Year group		
Year one students	39	33.6
Year two students	34	29.3
Year three students	43	37.1
Average age: 22 years		

### Procedures

A set of typical clinical scenarios from the radiation therapy setting was developed for each year of the programme during 2013. The level of skill required within the scenarios matched the development of students’ knowledge and clinical experience at each year level. Trained actors, as a standardised patient, staff or member of the public, role played the scenarios in a simulated high fidelity clinical setting. These actors have received formal training on what is required to portray a patient and staff member, and have had several years’ experience representing patients in clinical simulations. In addition, the actors were given a script of the context of each interaction: for patient‐focused scenarios information included cancer diagnosis, emotional and social issues, level of intensity. For qualified staff‐focused scenarios, information was provided on the collegial issue (involving professionalism and/or ethics) to be addressed in the scenario. Actors were given an opportunity to clarify what was required in each scenario with academic staff before the workshops.

Each student was assessed against six key communication skills, as outlined in Table 2. These skills were standardised communication skills that are taught in the academic healthcare communication and the clinical practice papers across the 3 years of the programme. Students were briefed on these skills prior to the workshops so that they knew what to expect.

**Table 2:** Key communication skills assessed across the 3 years of the programme

Year one	Year two	Year three
Initial engagement	Initial engagement	Initial engagement
Identifies the issue/s or concern/s	Identifies the issue/s or concern/s	Identifies the issue/s or concern/s
Imparts appropriate knowledge	Responds appropriately to situation	Exploration of issue/s or concern/s
Building the relationship	Building the relationship	Building the relationship
Appropriate action reached	Appropriate action reached	Reaching common ground
Closing interaction	Closing interaction	Closing interaction

In order to create a non‐threatening, confidential and safe learning environment, expectations of group behaviour and instructions for giving and receiving feedback were discussed with all students. Students were then divided into small groups accompanied by one staff member. The following proceeded:

1. Students were randomly allocated one clinical scenario according to their year level;
2. Students were briefed on the situation before entering a high fidelity clinical room and were required to manage their interactions appropriate to the context of their scenario;
3. Each student's interaction was observed via a digital screen by the small group and one staff member, who rated their observations of the student's performance on the six key skills and the open ended questions as stated in Table 3;

**Table 3:** Free text open ended questions for peer feedback

Question 1	What do you think your student peer did well?
Question 2	What do you think they need to improve?
Question 3	What did you learn from observing this scenario?
4. Each student was given their individual video recording for self‐review and reflection on their own performance in their own time. A set of written instructions was given to the students to guide their written responses.

A structured debriefing framework was used: (1) student self‐assessment, where each student was invited to defuse and then reflect on their interaction immediately after their scenario; (2) verbal feedback on performance by peers, actor and academic staff; (3) focused facilitation of student discussion by academic staff, experienced in group facilitation, to keep the learning environment safe; (4) collation of peer and academic staff assessment of performance according to the skills criteria; (5) collation of student written self‐reflections on their performance and perceptions of their learning; (6) a summary of the feedback was provided to each student. The purpose of this gap analysis was to identify discrepancies in student self‐perceptions of their performance, peer and staff assessments, to assist academic staff to address these with the student before their next clinical placement.

Qualitative data were obtained from the students’ written responses to the guided self‐reflection questions in Table 4.

**Table 4:** Free response qualitative questions

Question 1	What communication skills do you use/do well in your scenario? Please use examples of specific communication skills.
Question 2	What communication skills do you think you should improve on? Please use examples of specific communication skills.
Question 3	What would you do differently (if at all) in a similar situation?
Question 4	What did you learn from the verbal feedback given to you about your scenario?
Question 5	What have you learnt from observing your peers in this process?

Quantitative data were collected from student ratings of their experiences of the workshops, using a 5‐point Likert scale.

The two staff members examined the data independently to identify themes across the student responses. They then compared and discussed their independent findings to reach a consensus on the reoccurring themes.^24^

## Results

### Thematic analysis

Three main themes emerged from the data and were identified by the researchers as: the value of learning from peers; preparation for the clinical environment; and the ability to self‐reflect.

#### Learning from peers

The students appreciated observing their peers and the ensuing discussion about the scenarios. It appeared to help them realise that there is no one right way to manage interactions, as illustrated here:

It was really good seeing the range of responses others had to their scenarios. Hearing them talk about why they may have said or done a certain thing was really good/interesting to see. Everyone has different responses and that's okay.

Discussion with peers, the actor and academic staff was often highlighted as particularly useful, as stated here:

It was an awesome experience to observe how others discussed issues with patients especially the opportunity to talk about it afterwards with each other, a teacher and the ‘patient’.

I learnt the most from the discussion that we had as a group afterwards as I found the feedback from three different perspectives gave me more insight into what would be good ways to handle this sort of scenario.

Observational learning was helpful because it showed different approaches to managing interactions that students had not considered as an option. These findings also indicated that students liked the structured approach to debriefing as it appeared to help their learning.

#### Preparation for the clinical environment

Although some students felt confronted by the scenarios they also felt better prepared for the clinical environment because the scenarios were believable. Several students mentioned not only the challenges they faced in their scenario but also the relevance of it to the clinical arena, as illustrated by the following statements:

I froze up in the situation as well as trying to find a solution, found it very awkward but good practice as it could happen in the clinic and I feel more prepared now than I did before the situation.

This was the **most** helpful part. It gave me a way to reflect my own scenario and compare them. In general, it gave me a lot of helpful techniques that will certainly help me (mostly dealing with difficult people).

Some situations were very tough and I learnt the best/different ways to handle them through watching my peers. This was helpful for clinical as these situations can happen in real life. I think that although it is intimidating having peers watch in a tough situation, it is overall beneficial for us for clinical communication.

These findings suggest the value of utilising trained actors who can provide authenticity in common clinical scenarios, to enhance student engagement in their learning and better preparation for the clinical setting.

#### Self‐reflection

The findings indicate that students varied in their ability to self‐reflect on their performance, from minimal to more developed reflective skills. Irrespective of year groups, no difference within and between year groups was apparent.

The majority of students’ reflection focused on factual information only rather than reflection:

I think at the beginning I introduced myself well and tried to reassure the patient as much as possible that it was normal. This was to try and calm his nerves/anxiety.

Some students’ reflection showed understanding of their performance but with minimal reflection:

I think I could have tried to see what the patients’ problem was first and then worked together to move forward. I also need to watch what I say/the language that I used.

I could have done a much better job in showing empathy for the patient. I realised the concern he had, but did not identify it verbally, which made him more distressed as he could have felt he was not being heard. I also could have offered information about services that could help him with his concerns

Very few students demonstrated self‐reflection, as indicated by this comment:

When we are watching peers, we are separated and open, without any judgements. I think that this is actually a really good way to be when I find myself in difficult situations – to take a view of how I am acting from an open, non‐judgemental view, so that I can see what the issue is, and how to best deal with it in that moment.

The majority of students could identify their strengths and limitations within their own scenario, hence demonstrating self‐awareness. However, the ability to self‐reflect did not appear to develop alongside this self‐awareness.

### Student perceptions

**Figure 1:**
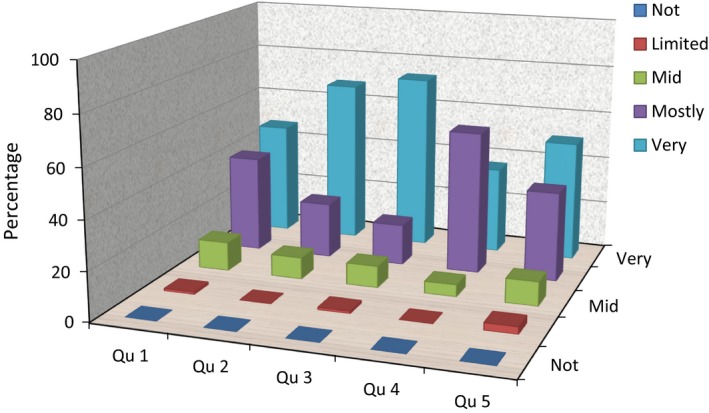
Overall student evaluations of their experience. Qu 1: How useful did you find participating in the communication scenario as a learning tool? Qu 2: How useful did you find observing peers in their communication scenarios? Qu 3: How effective were the actors in engaging you in the scenario? Qu 4: How comfortable were you when receiving verbal feedback about your scenario from peers, staff and actors? Qu 5: Overall, how effective were the communication scenarios as a learning tool?

In 2014 the survey of student perceptions was added to the student self‐reflection form so 78/116 participants completed the Likert scale questionnaire detailed in Figure 1.

Figure 1 indicates that overall most the students across the 3 years of the BRT programme found the communication workshops to be a useful, valuable and authentic learning experience. Some students, however, are not yet comfortable receiving feedback as highlighted by the predominately mostly comfortable response in question four.

## Discussion

This innovative study in the radiation therapy setting aimed to explore students’ perceptions of their learning from participation in communication skills workshops. Overall, the students reported that participating in these workshops was a positive learning experience. The students valued observing differences in peer interactions and witnessing the impact of effective and ineffective strategies to manage various clinical situations. This is consistent with Bandura's social learning theory^18^ that emphasises the importance of observing others, cognitive rehearsal of witnessed behaviours, followed by deliberate practice to reinforce learning, as pivotal aspects of the learning process. Similarly, learning from peers also corresponds with Vygotsky's zone of proximal development (ZDP)^22^ in which learners bring their current understandings to the simulated scenario. New ideas are introduced and then linked to students’ existing knowledge. Hence, observational and active learning have the capacity to extend the students’ ZDP in relation to their communication skills. This gives students an opportunity to cognitively prepare for similar professional situations in the clinical setting. Currently no time is allocated for deliberate practice to further embed the learning gained, due to financial and timetabling constraints in the radiation therapy programme. However, communication skills do not readily lend themselves to deliberate practice because there is no single way to respond to interactions; each context influences what is appropriate. Since the students participate in the communication workshops across the 3 years of the programme, there is opportunity provided for *reflection on practice* as part of their ongoing development.

Authenticity and fidelity are essential for the scenario to be believable. The students highly rated these aspects of the scenarios as evidenced in Figure 1. The use of trained actors as standardised patients, staff or members of the public, was effective. The actors brought psychological, emotional, and social nuances to the simulated scenarios, with differing levels of intensity. This helped the students to suspend disbelief. Another important role of the actors was to coach the students by giving feedback on the student's demeanor, such as their introduction, non‐verbal behaviours, quality of listening, emotional and relational aspects of the interactions. The actors also highlighted moments in the interactions that stood out for them and explained why. The actors’ feedback also facilitated student reflection on the process through gaining insight into the experience of patient, staff or member of the public because students do not usually receive this information from ‘real’ people in the clinical environment. This was meaningful to the students and they saw how this could be of value in the clinical environment. This reinforces the value of utilising trained, skilled actors, which has been well documented in the literature to provide high fidelity and quality coaching.^6^, ^10^, ^25^, ^26^

In order for simulation to be a positive learning experience, it was essential to create a safe learning environment in which students felt secure that their participation was valued. This is in line with recommendations offered by Pascucci et al.,^26^ who reiterate the importance of providing a safe learning environment. The structured facilitation and debriefing process also reinforced the principles of respect and confidentiality. This was mostly successful as highlighted in the data in Figure 1 where students rated that they felt mostly or very comfortable with the process. To address some of the safety issues, participation in the simulated scenarios is a compulsory formative exercise, so students are not graded on their own performance. This is the real strength of the experience that students value. Therefore, managing student vulnerability in simulation needs to be high on academic staff's agenda to enable learning, including self‐reflection.

According to Schön^27^ individuals need to make sense of new information and how they might integrate and implement this knowledge. Hence, self‐reflection was embedded in the process of reflecting in and on practice during the structured debriefing and guided written self‐reflection. The aim of which was to assist students to integrate knowledge gained from simulation, which Schön refers to as *reflection on action*. This method is consistent with Eppich and Cheng's^28^ scripted debriefing approach to providing feedback which starts with learner defusing; describing their experience; followed by analysis of how the situation was managed. Similarly, peers, actors and then staff provide feedback according to the same process, followed by a facilitated discussion that highlights the professional issue in the situation. Directive instruction is only provided when it is clear that the students have misunderstood the issue(s) involved. This structured debriefing approach was utilised in our study and appeared to be effective from the students’ perspective. Students valued hearing feedback from these different perspectives, in particular from the actors. While our debriefing was teacher‐led, what is not yet clear is whether this was an effective model to use. As Issenberg et al.^15^ have highlighted, limited research has investigated whether peer‐led, staff‐led or self‐review is the most effective debriefing model to use.

Self‐reflection is a critical clinical skill that students find challenging to develop. While the purpose of our study was to examine students’ perceptions of their learning from participating in simulated scenarios, a secondary finding was that students demonstrated variable levels of reflection at each year level, as evidenced in their written reflections. The majority of students showed some understanding of their performance regarding what worked and what did not work, but lacked deeper insight on the strategies they used and whether these were effective or not. There was a sub‐group of students who did not show any reflection and merely identified the micro‐skills used, and were not able to analyse why these worked or not. Even fewer students demonstrated in‐depth self‐awareness in their reflections. Our data therefore suggest that students have self‐awareness as demonstrated in their own self‐assessment of communication skills. However, this self‐awareness does not appear to transfer to reflection; more structured guidance on the process of reflection is warranted. While self‐reflection is embedded in both the academic and clinical papers, it is obvious from these findings that students struggle with self‐reflection. It is unclear whether this is a maturational aspect of the student body who are mostly young adults under 24 years old and have had limited clinical experience. Further research needs to examine the processes of *reflecting in* and *on practice*, as pointed out by Issenberg et al.^15^

According to the quantitative data, student perceptions of the tool are positive and they rate it as an effective learning experience. In line with Issenberg et al.^15^, these findings suggest that the tool is robust from the students’ point of view. Despite positive student ratings of the tool, the findings also highlight that students find receiving verbal feedback challenging, as indicated in Question 4, Figure 1. Preparing students to receive feedback during the debriefing needs further addressing in order to foster a safer learning environment. This could be for a range of reasons: students are randomly assigned to groups with peers they may not get on well with and so may anticipate very critical feedback or not take on‐board their feedback; they may fear being observed by the group and have performance anxiety so it may not be a true reflection of their ability; being video recorded is confronting and so they may be overly conscious of the recording equipment, their peer and academic staff observers.

### Limitations

All of the data has been collected via student self‐report. There may be some bias in how students rate their experience in order to please the teaching staff; and this may also be a consideration in their reflections.

Peer evaluations pose some difficulties in interpretation because of the Likert ratings do not really reflect the verbal and written feedback. This indicates that students do not seem to enjoy formally rating their peer's performance.

Follow‐up is undertaken with the small number of students who do not demonstrate self‐awareness of their overall performance and coaching is provided before they enter the clinical environment. However, feedback about the benefits of coaching was not solicited.

## Conclusion

This is the only study in radiation therapy training that examines students’ perceptions of participation in communication skills workshops. Students perceive the scenarios to be helpful for their learning. High fidelity, authentic simulated clinical scenarios, with the integration of trained actors are a valuable approach to assist radiation therapy students to develop their communication skills. A structured debriefing process is a beneficial approach to giving and receiving feedback within a safe learning environment. However, while students demonstrate self‐awareness, this does not appear to transfer into reflection; most of their written self‐reflections show understanding of the situation but not *reflection on action*. This warrants further investigation, with a specific focus on developing a more structured approach to guiding student *reflection on action* during the debriefing. Further research will examine whether improvements in the debriefing approach to reflection helps to develop this skill.

## References

[1] Medical Radiation Technologists Board . Code of Ethics for Medical Radiation Technologists [Internet]. New Zealand: 2004 [cited 15 June 2015]. Available from: http://www.mrtboard.org.nz/publications/.

[2] Issenberg S , Scalese R . Simulation in health care education. Perspect Biol Med 2008; 51: 31–46. 18192764 10.1353/pbm.2008.0004

[3] Bland AJ , Topping A , Wood B . A concept analysis of simulation as a learning strategy in the education of undergraduate nursing students. Nurse Educ Today 2011; 31: 664–70. 21056920 10.1016/j.nedt.2010.10.013

[4] Lateef F . Simulation‐based learning: Just like the real thing. J Emerg Trauma Shock 2010; 3: 348–52. 21063557 10.4103/0974-2700.70743 PMC2966567

[5] LeBlanc VR . Review article: Simulation in anesthesia: State of the science and looking forward. Can J Anesth 2012; 59: 193–202. 22179792 10.1007/s12630-011-9638-8

[6] Marken P , Zimmerman C , Kennedy C , Schremmer R , Smith KV . Human simulators and standardized patients to teach difficult conversations to interprofessional health care teams. Am J Pharm Educ 2010; 74: 1–8. 21088725 10.5688/aj7407120 PMC2972514

[7] Galal S , Carr‐Lopez S , Seal CR , Scott AN , Lopez C . Development and assessment of social and emotional competence through simulated patient consultations. Am J Pharm Educ 2012; 76: 1–7. 23049104 10.5688/ajpe767132 PMC3448470

[8] Herge EA , Lorch A , Deangelis T , Vause‐Earland T , Mollo K , Zapletal A . The standardized patient encounter: A dynamic educational approach to enhance students’ clinical healthcare skills. J Allied Health 2013; 42: 229–35. 24326920

[9] Webster D , Seldomridge L , Rockelli L . Making it real: Using standardized patients to bring case studies to life. J Psychosoc Nurs Ment Health Serv 2012; 50: 36–41. 10.3928/02793695-20120410-06 22533843

[10] Rutherford‐Hemming T , Jennrich J . Using standardized patients to strengthen nurse practitioner competency in the clinical setting. Nurs Educ Perspect 2013; 34: 118–21. 23763026 10.5480/1536-5026-34.2.118

[11] Keltner NL , Grant JS , McLernon D . Use of actors as standardized psychiatric patients: Facilitating success in simulation experiences. J Psychosoc Nurs Ment Health Serv 2011; 49: 34–40. 10.3928/02793695-20110329-02 21485978

[12] Doody O , Condon M . Using a simulated environment to support students learning clinical skills. Nurse Educ Pract 2013; 13: 561–6. 23602694 10.1016/j.nepr.2013.03.011

[13] Rush S , Firth T , Burke L , Marks‐Maran D . Implementation and evaluation of peer assessment of clinical skills for first year student nurses. Nurse Educ Pract 2012; 12: 219–26 A. 22357193 10.1016/j.nepr.2012.01.014

[14] Baillie L , Curzio J . Students’ and facilitators’ perceptions of simulation in practice learning. Nurse Educ Pract 2009; 9: 297–306. 18842463 10.1016/j.nepr.2008.08.007

[15] Issenberg SB , Ringsted C , Østergaard D , Dieckmann P . Setting a research agenda for simulation‐based healthcare education a synthesis of the outcome from an utstein style meeting. Simul Healthc 2011; 6: 155–67. 21642804 10.1097/SIH.0b013e3182207c24

[16] Kolb DA . Experiential Learning: Experience as the Source of Learning and Development. 1 edn Prentice Hall, New Jersey, 1984.

[17] Burke H , Mancuso L . Social cognitive theory, metacognition, and simulation learning in nursing education. J Nurs Educ 2012; 51: 543–8. 22909039 10.3928/01484834-20120820-02

[18] Bandura A . Social cognitive theory : An agentic perspective. Annu Rev Psychol 2001; 52: 1–26. 11148297 10.1146/annurev.psych.52.1.1

[19] Fanning RM , Gaba DM . The role of debriefing in simulation‐based learning. Simul Healthc 2007; 2: 115–25. 19088616 10.1097/SIH.0b013e3180315539

[20] Miles L , Mabey L , Leggett S , Stansfield K . Teaching communication and therapeutic relationship skills to baccalaureate nursing students: A peer mentorship simulation approach. J Psychosoc Nurs Ment Health Serv 2014; 52: 34–41. 10.3928/02793695-20140829-01 25207556

[21] Dannefer EF , Henson LC , Bierer SB , et al. Peer assessment of professional competence. Med Educ 2005; 39: 713–22. 15960792 10.1111/j.1365-2929.2005.02193.x

[22] Vygotsky L . Interaction between learning and development In: Gauvin M Cole M (eds). Readings on the Development of Children. W.H. Freeman and Company, New York, 1997; 29–36.

[23] Roh YS , Issenberg SB , Chung HS , Kim SS , Lim TH . A survey of nurses’ perceived competence and educational needs in performing resuscitation. J Contin Educ Nurs 2013; 44: 230–6. 23458080 10.3928/00220124-20130301-83

[24] Braun V , Clarke V . Using thematic analysis in psychology. Qual Res Psychol 2006; 3: 77–101.

[25] Davis AH , Kimble LP , Gunby SS . Nursing faculty use of high‐fidelity human patient simulation in undergraduate nursing education: A mixed‐methods study. J Nurs Educ 2014; 53: 142–50. 24530129 10.3928/01484834-20140219-02

[26] Pascucci RC , Weinstock PH , O'Connor BE , Fancy KM , Meyer EC . Integrating actors into a simulation program. Simul Healthc 2014; 9: 120–6. 24096918 10.1097/SIH.0b013e3182a3ded7

[27] Schőn DA . The Reflective Practitioner: How Professionals Think In Action. 1st edn Basic Books, USA, 1983.

[28] Eppich W , Cheng A . Promoting Excellence and Reflective Learning in Simulation (PEARLS). Simul Healthc 2015; 10: 106–15. 25710312 10.1097/SIH.0000000000000072

